# Projecting the impacts of rising seawater temperatures on the distribution of seaweeds around Japan under multiple climate change scenarios

**DOI:** 10.1002/ece3.1358

**Published:** 2014-12-18

**Authors:** Shintaro Takao, Naoki H Kumagai, Hiroya Yamano, Masahiko Fujii, Yasuhiro Yamanaka

**Affiliations:** 1 Faculty of Environmental Earth Science, Hokkaido UniversityNorth 10 West 5, Kita-ku, Sapporo, Hokkaido, 060-0810, Japan; 2 Center for Environmental Biology and Ecosystem Studies, National Institute for Environmental Studies16-2 Onogawa, Tsukuba, Ibaraki, 305-8506, Japan

**Keywords:** Barren ground, climate change, future projection, global warming, herbivores, seaweed

## Abstract

Seaweed beds play a key role in providing essential habitats and energy to coastal areas, with enhancements in productivity and biodiversity and benefits to human societies. However, the spatial extent of seaweed beds around Japan has decreased due to coastal reclamation, water quality changes, rising water temperatures, and heavy grazing by herbivores. Using monthly mean sea surface temperature (SST) data from 1960 to 2099 and SST-based indices, we quantitatively evaluated the effects of warming seawater on the spatial extent of suitable versus unsuitable habitats for temperate seaweed *Ecklonia cava,* which is predominantly found in southern Japanese waters. SST data were generated using the most recent multiple climate projection models and emission scenarios (the Representative Concentration Pathways or RCPs) used in the Coupled Model Intercomparison Project phase 5 (CMIP5). In addition, grazing by *Siganus fuscescens,* an herbivorous fish, was evaluated under the four RCP simulations. Our results suggest that continued warming may drive a poleward shift in the distribution of *E. cava,* with large differences depending on the climate scenario. For the lowest emission scenario (RCP2.6), most existing *E. cava* populations would not be impacted by seawater warming directly but would be adversely affected by intensified year-round grazing. For the highest emission scenario (RCP8.5), previously suitable habitats throughout coastal Japan would become untenable for *E. cava* by the 2090s, due to both high-temperature stress and intensified grazing. Our projections highlight the importance of not only mitigating regional warming due to climate change, but also protecting *E. cava* from herbivores to conserve suitable habitats on the Japanese coast.

## Introduction

Climate change is expected to alter species distribution ranges (e.g., Hoegh-Guldberg and Bruno 2010). Although long-term studies examining the effects of climate change on marine species, in comparison with terrestrial species, remain scarce (Rosenzweig et al. 2008), several reports have documented distributional shifts/expansions for several species, including seaweeds (e.g., Wernberg et al. 2011; Tanaka et al. 2012), corals (e.g., Yamano et al. 2011), and fishes (e.g., Perry et al. 2005). Furthermore, the distributional limits are more closely related to thermal tolerance limits in marine species compared with terrestrial species (Sunday et al. 2012).

Among the most clear and profound influences of climate change on the world's oceans are its impacts on habitat-forming species (Hoegh-Guldberg and Bruno 2010). Seaweeds play a key role in providing essential habitats and energy for thousands of associated organisms (Dayton 1985). Therefore, their loss or replacement by other species due to climate change could have major implications for biodiversity, ecological function, biogeochemical cycling, and human society.

Japan is uniquely suited for assessing distributional shifts in seaweed populations due to climate change for several reasons. First, Japan covers a wide latitudinal range, stretching from subtropical to temperate areas. Second, the Japanese islands form an almost continuous chain, such that subtropical and warm temperate seaweeds can inhabit the extent of this latitudinal gradient, due to the warm Kuroshio Current and its branches (e.g., Okuda 2008). Third, seawater temperatures are rising more rapidly around Japan than in other parts of the world's oceans (Japan Meteorological Agency 2013). Average sea surface temperatures (SSTs) around Japan have risen by +1.08°C per century from 1891 to 2012, which is double the corresponding value for the world's oceans (+0.51°C per century). Finally, seaweeds around Japan have been observed and documented periodically since the mid 20th century (e.g., Tanaka et al. 2012; Data S1), providing a much-needed baseline against which to assess the impacts of rising water temperatures on their distribution.

Japanese coastal areas are also a global biodiversity hot spot (Fujikura et al. 2010; Tittensor et al. 2010). In fact, 14.6% of marine organisms identified around the world are living within these areas (Fujikura et al. 2010). Based on survey data, the total area of seaweed beds throughout coastal Japan decreased from 2012 km^2^ in 1989–1991 to 1425 km^2^ in 1998 (The Environmental Agency of Japan 1994, 1998). The largest decrease in habitat-forming seaweeds has been observed for temperate kelps (Fujita 2010), and the greatest causes of this decline have been attributed to coastal reclamation, water quality changes, increased seawater temperatures, and grazing by herbivores, which can lead to the formation of “barren ground” (e.g., Okuda 2008). Barren ground, a phenomenon referring to the total disappearance of seaweeds, is a serious problem for Japanese coastal areas (Fisheries Agency of Japan 2007). Rising seawater temperatures can affect seaweeds, either directly through physiological impacts (e.g., a negative effect on photosynthesis) (Serisawa et al. 2001; Tanaka et al. 2008) or indirectly via prolonged grazing by herbivorous fish (Yamaguchi et al. 2010). For these reasons, barren ground events are expected to become more frequent and severe around Japan due to ongoing regional warming.

The species *Ecklonia cava*, a temperate kelp, predominates throughout the southern coast of Japan and can be found among a wide range of surface water temperatures above 10°C (Suto 1992). In this region, *E. cava* is also commercially important, given that it is a primary food source for shellfish, such as abalone, and thus provides economic support for fisheries (Nonaka and Iwahashi 1969). However, *E. cava* populations have declined rapidly since the 1990s, and several populations disappeared entirely from the coasts of southwestern Japan by 2000 (e.g., Serisawa et al. 2004; Haraguchi et al. 2009; Tanaka et al. 2012; Kiyomoto et al. 2013). The decline of *E. cava*, which leads to the barren ground phenomenon, can be attributed to rising seawater temperatures along with heavy grazing by sea urchins and herbivorous (omnivorous, but seaweed preferring) fishes, such as *Siganus fuscescens* (Masuda et al. 2000, 2007; Serisawa et al. 2004; Haraguchi et al. 2009). The loss of *E. cava* may subsequently drive secondary disruptions in associated ecosystems, which may explain the recent decrease in the annual landing of abalone in southwestern Japan (Serisawa et al. 2004; Kiyomoto et al. 2013). However, our understanding of how continued warming will affect *E. cava* populations and habitat over the coming decades remains uncertain. Therefore, long-term future projections of the effects of climate change on distributional shifts in *E. cava* habitat, derived from climate models, are crucial to designing measures for the conservation of marine biodiversity and the adaptation of human societies to coming changes. Furthermore, given that grazing by herbivores could potentially compound the direct effects of rising seawater temperatures on *E. cava* populations, we must distinguish which habitats of *E. cava* will be affected by high temperatures and/or heavy grazing. Such zoning will be useful for designing interventions for conservation and adaptation.

Distributional shifts in seaweeds in response to future seawater temperature increases have been projected by previous studies (Müller et al. 2009; Jueterbock et al. 2013; Raybaud et al. 2013). However, most of these studies focused solely on the Atlantic Ocean and used only one or two models with multiple emission scenarios (Jueterbock et al. 2013; Raybaud et al. 2013) or models with only one emission scenario (Müller et al. 2009). Furthermore, only Raybaud et al. (2013) have reported future projections on the distributional shifts of seaweeds using the most recent climate models of the Coupled Model Intercomparison Project phase 5 (CMIP5; Taylor et al. 2012), which was performed for the Fifth Assessment Report of the Intergovernmental Panel on Climate Change (IPCC AR5; Stocker et al. 2013).

In this study, for the first time, distributional shifts in the habitat of *E. cava* around Japan were assessed with regard to rising seawater temperatures over the course of the 21st century. This analysis was based on SST outputs provided by multiple CMIP5 climate model projections forced with all four future emissions scenarios of the Representative Concentration Pathways (RCPs; Moss et al. 2010). To obtain more realistic habitat projections for *E. cava*, a database of past-to-present *E. cava* distributions was assembled and used to validate potential habitats estimated from the modeled SSTs. Furthermore, where previous studies using climate model projections have focused on the impacts of rising water temperatures on seaweed distribution (Müller et al. 2009; Jueterbock et al. 2013; Raybaud et al. 2013), we also evaluated the effects of grazing by the herbivorous fish *S. fuscescens* on the distribution of *E. cava* under various warming scenarios. To the best of our knowledge, projections such as these, which consider the interspecific interaction between seaweeds and herbivores, have never before been published. Thus, our findings fill an important gap in the literature given that the loss of an ecosystem engineer, such as *E. cava*, may lead to cascading effects throughout the coastal marine ecosystem.

## Materials and Methods

### Presence–absence data for *E. cava*

Successive distributional records with a broad regional range and covering decadal time scales are vital for assessing distributional shifts in marine species and their processes in response to recent warming (Tanaka et al. 2012). Although comprehensive database of the distribution of *E. cava* populations has been created previously (Terawaki et al. 1991; Terada et al. 2013), there is no time-series database thus far. Therefore, we collected distributional data derived from the literature and report for *E. cava* in Japanese coastal waters (Data S1) and compiled them in chronological order. In this study, data for *E. cava* were combined with those for *E. kurome*, which is regarded as the taxonomic synonym of *E. cava*, assuming that no genetic difference exists between the species (Tanaka et al. 2007). The compiled records provided coverage from the 1960s to 2000s. We then analyzed chronological changes in the distribution of *E. cava* for each of 20 cells comprising a 1°×1° resolution grid (“grid cell” hereafter), where historical records were available (Fig. S1) over a decadal time scale (i.e., 10-year intervals), to validate the potential habitats estimated from modeled SSTs and simplified indices (see below). Presence of *E. cava* at each grid cell was assigned if 50% or more than 50% of the existing records cited the species. A grid cell with no distributional record for *E. cava* was assigned via temporal interpolation where possible. For example, if a grid cell with no record in 1980s was found to contain *E. cava* before the 1970s and after the 1990s, the grid cell was assumed to similarly contain *E. cava* in the 1980s. However, if no previous information on the presence/absence of *E. cava*, the grid cell was regarded to have no data. In addition, when the modeled grid cells were located offshore relative to the distributional records, adjacent coastal data near the grid cell were used to maximize the available dataset. The numbers of records used to validate the estimated potential habitats were summarized in Table S1.

### Datasets of modeled sea surface temperatures

Ensembles of climate models were used to estimate potential habitats for *E. cava* and the likelihood of year-round grazing areas by the herbivorous fish *S. fuscescens,* based on the SST-based indices described below. In this study, we used 17 CMIP5 model projections (Table1), which were available for historical simulations and four RCP simulations (i.e., RCP2.6, RCP4.5, RCP6.0, and RCP8.5). The RCPs produced were those that will lead to radiative forcing levels of 2.6, 4.5, 6.0, and 8.5 W/m^2^ by the end of the 21st century with the globally averaged mole fractions of CO_2_ reaching 421, 538, 670, and 936 ppm, respectively (van Vuuren et al. 2011). Monthly SSTs from the model projections were obtained from the Program for Climate Model Diagnosis and Intercomparison (http://cmip-pcmdi.llnl.gov/cmip5/availability.html). We used the historical simulations from 1960 to 2005 and the climate change projections from 2006 to 2099 under the four RCP simulations. Although there are generally biases and errors in climate model results, using the averaged result from multiple climate models can improve the performance of a forecast and decrease uncertainties in the result (Tebaldi and Knutti 2007). Thus, the SSTs obtained using the 17 CMIP5 models were averaged in this study. To correct biases and compute model means, the modeled SSTs were interpolated to a regular 1°×1° grid cell using the nearest neighbor method.

**Table 1:** List of climate models used in this study, horizontal resolution, and ocean model

Model (Country)	Horizontal resolution (Longitude × Latitude)	Ocean model
1. BCC-CSM1-1 (China)	1° ×0.33-1°	MOM4_L40
2. BCC-CSM1-1m (China)	1° × 0.33–1°	MOM4_L40
3. CCSM4 (USA)	1.125° × 0.27–0.54°	POP2.0
4. CESM1-CAM5 (USA)	1.125° × 0.27–0.53°	POP2.0
5. CSIRO-Mk3.6.0 (Australia)	1.875° × 0.9375°	MOM2.2
6. FIO-ESM (China)	1.125° × 0.27–0.53°	POP2.0
7. GFDL-CM3 (USA)	1° × 0.33–1°	MOM4p1
8. GFDL-ESM2G (USA)	1° × 0.375–1°	GOLD
9. GFDL-ESM2M (USA)	1° × 0.33–1°	MOM4p1
10. GISS-E2-H (USA)	2.5° × 2°	Hycom
11. GISS-E2-R (USA)	2.5° × 2°	Russell
12. HadGEM2-ES (UK)	1° × 0.34–1°	HadGOM2.0
13. IPSL-CM5A-LR (France)	2° × 0.5–2°	NEMO v2.3
14. MIROC5 (Japan)	1.406° × 0.5–1.378°	COCO v4.5
15. MRI-CGCM3 (Japan)	1° × 0.5°	MRI.COM3
16. NorESM1-M (Norway)	1.125° × 0.27–0.54°	MICOM
17. NorESM1-ME (Norway)	1.125° × 0.27–0.54°	MICOM

Biases in the monthly SST from each CMIP5 model were corrected using observed monthly data (1986–2005) of the National Oceanic and Atmospheric Administration (NOAA) Optimum Interpolation SST (OISST) version 2 (Reynolds et al. 2007), which were provided by the NOAA/OAR/ESRL PSD (Boulder, CO, http://www.esrl.noaa.gov/psd/). Although the OISST data were composed of SST obtained from in situ measurements as well as satellite-derived SST data and SST simulated by satellite-derived sea-ice cover, many modeling studies have regarded and used as observed SST data thus far (e.g., Yara et al. 2011, 2014; van Hooidonk et al. 2014). The bias-correction procedure was detailed in Yara et al. (2011, 2014). Briefly, we first calculated monthly SST anomalies during the 1960–2099 period (i.e., 1680 months) in the historical and RCP simulations relative to monthly climatology data (the 20-year mean SST from 1986 to 2005) in the historical simulation. Second, the calculated monthly SST anomalies for each year during the 1960–2099 period (i.e., monthly data for 140 years) were added to the monthly mean of the OISST 1986–2005 climatology data (i.e., monthly data), interpolated to a 1°×1° grid cell in the same manner as the CMIP5 models. Then, SSTs during the warmest/coldest months of each year were calculated from the bias-corrected SSTs for the period spanning 1960–2099. Finally, to simulate potential habitats for *E. cava*, we computed the CMIP5 multimodel monthly SST mean in the warmest/coldest months of each year spanning 1960–2099, using the bias-corrected SSTs of the warmest/coldest months from the 17 CMIP5 models.

### SST-based indices for *E. cava* distribution

Previous field studies have reported that *E. cava* cannot maintain populations in waters in which the annual minimum seawater temperature is below 10°C (Suto 1992). Thus, in this study, a suitable habitat for *E. cava* was defined as an area in which SSTs during the coldest month exceeded 10°C every year of the decadal timescales evaluated. In other words, when once SST in the coldest month below 10°C occurs within a decadal period (e.g., 1960s), *E. cava* populations cannot establish its suitable habitat. Regarding the effects of high-temperature stress, few studies have reported tolerable temperatures for *E. cava* populations based on laboratory experiments (e.g., Serisawa et al. 2001; Tanaka et al. 2008). Those studies have reported that the growth of sporophytes of *E. cava* increased with temperature from 10 to 27°C. In warmer water, Serisawa et al. (2001) have reported the growth decreased rapidly with temperature, and Tanaka et al. (2008) have reported that many gametophytes of the species grew abnormally at 28°C. Thus, in this study, we assumed that *E. cava* populations cannot establish its suitable habitat when SST in the warmest month exceeded 28°C more than once within a decadal period. To verify this hypothesis, we examined the number of times that the upper thermal threshold of 28°C exceeded per decade to maximize model performance for projecting *E. cava* habitats, by referring to the observed chronological change in the presence/absence of *E. cava* from the 1960s to 2000s (Table S1). For example, a threshold of 0.4 indicates that SST in the warmest month exceeded 28 °C occurs four times a decade. Model performance was assessed using correct projection rate (which assesses agreement between the observed and modeled data) and the receiver operating characteristic curve (ROC). The ROC is sensitivity (rate of true positive projection) as a function of 1 minus specificity (specificity is the rate of true negative projection) at various settings. The area under ROC (AUC) provides a single value representing model accuracy, and. AUC values closer to one indicate a better fit between the observed and modeled data (Jueterbock et al. 2013). In this study, the point at which sensitivity and specificity were equally maximized was chosen as the threshold (Liu et al. 2005).

### SST-based index for *S. fuscescens* grazing

**Figure 1:**
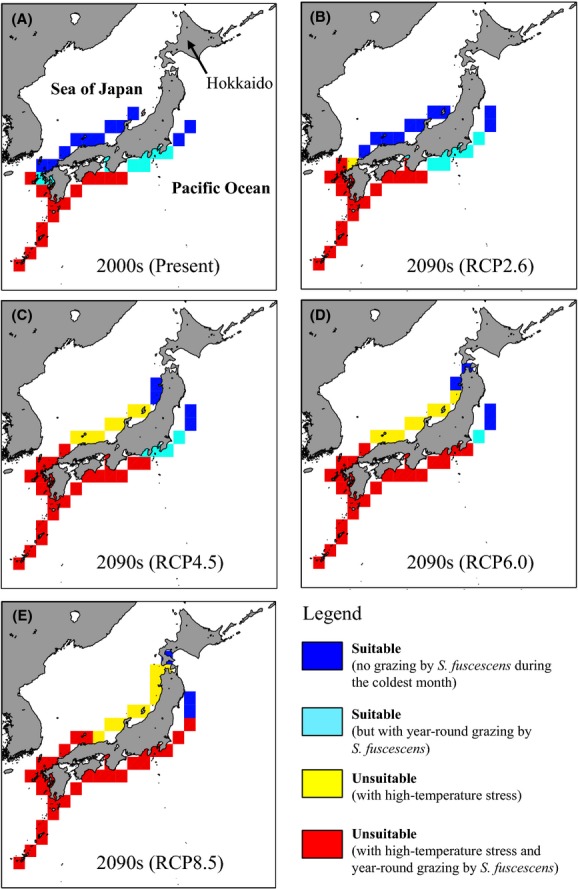
Potential habitats for *Ecklonia cava* projected using the CMIP5 multimodel mean monthly SSTs in (A) the 2000s and (B–E) the 2090s for RCPs 2.6, 4.5, 6.0, and 8.5, respectively. The results in the 2000s were projected based on a historical simulation for 2000–2005 and the mean of the four RCP simulations for 2006–2009. Blue squares, suitable habitats without high-temperature stress or year-round grazing by *S. fuscescens*; light blue squares, suitable habitats without high-temperature stress but with year-round grazing; yellow squares, unsuitable habitats due to high-temperature stress; red squares, unsuitable habitats due to a combination of high-temperature stress and year-round grazing.

Rising water temperatures affect distribution ranges of *E. cava* not only directly, via adverse physiological responses*,* but also indirectly, via the intensification of grazing. Grazing by the herbivorous fish *S. fuscescens* has been identified as one of the potential causes of *E. cava* decline around Japan (Masuda et al. 2000, 2007; Kiriyama 2009). Although *S. fuscescens* has been found in all Japanese coastal areas except Hokkaido (Fujita 2010; Fig.1A), it is difficult to explain the decline of *E. cava* populations by only the distribution of the herbivorous fish species. Thus, in this study, we focused on the changes in year-round grazing area by *S. fuscescens* in warming water rather than those in their distribution. The grazing of this species on *E. cava* populations is dependent on ambient water temperatures (Yamauchi et al. 2006). In a laboratory experiment, Yamauchi et al. (2006) reported that *S. fuscescens* grazed *E. cava* populations intensively under warmer temperatures (26–29°C), whereas grazing was low in temperatures below 20°C and minimal below 15°C. Thus, in this study, the year-round grazing territories of *S. fuscescens* were defined as the areas in which the mean monthly SST during the coldest month was higher than 15°C each year.

## Results

### Assessing model performance

Assessments of the threshold maximized the projected probability of the *E*. *cava* habitat are summarized in Table2. The threshold maximized the projected probability was chosen to maximize sensitivity and specificity equally. We found that both sensitivity and specificity were higher when the threshold took 0.3 or 0.4 (i.e., the disappearance of *E. cava* populations due to high-temperature stress occurs more than three or four times per decade, respectively). Among the other parameters used to assess model performance, both the AUC and correct projection rate were highest when using a threshold of 0.4 (Table2). These results indicate that the modeled change in potential habitats was in good agreement with the observed chronological change when areas in which the disappearance of *E. cava* populations due to high-temperature stress occurred more than four times per decade were defined as unsuitable habitats. Thus, all subsequent results were based on projections using a threshold of 0.4.

**Table 2:** Assessment of the threshold maximized the projected probability of *Ecklonia cava* habitats. AUC, area under a receiver operating characteristic curve; correct, agreement rate between the observation and the projection; sensitivity, rate of true positive projection; specificity, rate of true negative projection

Threshold	AUC	Correct	Sensitivity	Specificity
0.1	0.802	0.823	0.855	0.750
0.2	0.779	0.823	0.891	0.667
0.3	0.776	0.835	0.927	0.625
0.4	0.785	0.848	0.945	0.625
0.5	0.773	0.848	0.964	0.583
0.6	0.783	0.861	0.982	0.583
0.7	0.762	0.848	0.982	0.542

### Potential habitats for *E. cava* under warming scenarios

**Figure 2:**
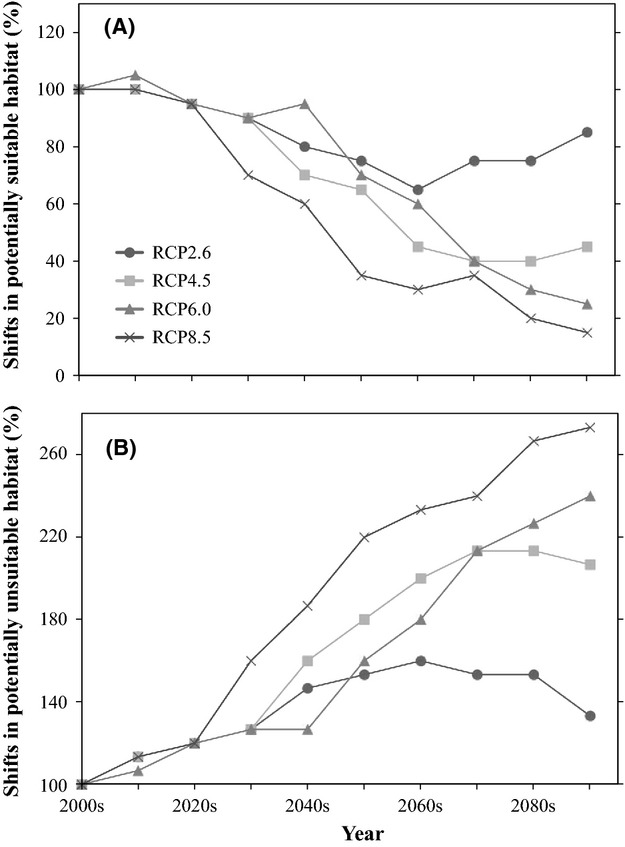
Shifts in the spatial extent of potentially (A) suitable and (B) unsuitable habitats for *Ecklonia cava* from 2000s to 2090s.

Figure1 shows the suitable habitats for *E. cava* projected using the CMIP5 multimodel monthly mean SST from 2000s to 2090s. We observed significant shifts in the potential habitats for *E. cava* depending on the warming scenario examined (Figs1 and S2). For the RCP2.6 scenario, little difference was observed in the northern and southern habitat limits between 2000s and 2090s (Fig.1A,B). Compared with the present, although there was no latitudinal shift of the suitable habitat for *E. cava* by the 2090s (Table3), the total area of the suitable habitat was projected to decrease by 15% in the 2090s, whereas unsuitable habitat was simulated to increase by 33% (Fig.2). For the other warming scenarios, however, potentially suitable habitats shifted to higher latitudes (i.e., poleward) in the 2090s, and the projected speeds of the poleward expansion for *E. cava* were 2.2, 3.3, and 4.5 km/year by the 2090s under RCPs 4.5, 6.0, and 8.5, respectively (Table3). On the other hand, the suitable habitat was reproduced to decrease in total spatial extent compared with that available during the 2000s, which was due to rising water temperatures and subsequent disappearance of *E. cava* populations (Fig.1C–E). Changes in suitable habitat were relatively constant until the 2020s for all RCPs. However, the spatial extent of suitable habitats decreased dramatically thereafter and were half of the present level in the 2060s, 2070s, and 2050s under RCPs 4.5, 6.0, and 8.5, respectively (Fig.2A). Finally, the spatial extent of suitable habitats decreased to 45%, 25%, and 15% of the present extent by the 2090s under RCPs 4.5, 6.0, and 8.5, respectively. At the same time, unsuitable habitats in the RCP4.5 scenario doubled by the end of the 21st century and that for the RCP8.5 nearly tripled (Fig.2B).

**Table 3:** Projected speeds of the poleward expansion of the suitable habitat for *Ecklonia cava* under the four RCP scenarios. The speeds were calculated from differences in the northern position of the habitats between 2000s and 2090s

	Projected speeds of the poleward expansion of the suitable habitat for *E. cava* [km/year]	Northern position of the suitable habitat for *E. cava* in 2000s [°N]	Northern position of the suitable habitat for *E. cava* in 2090s [°N]
RCP2.6	0	37.5	37.5
RCP4.0	2.2	37.5	39.5
RCP6.0	3.3	37.5	40.5
RCP8.5	4.5	37.5	41.5

### Effects of herbivorous fish on potential habitats

**Figure 3:**
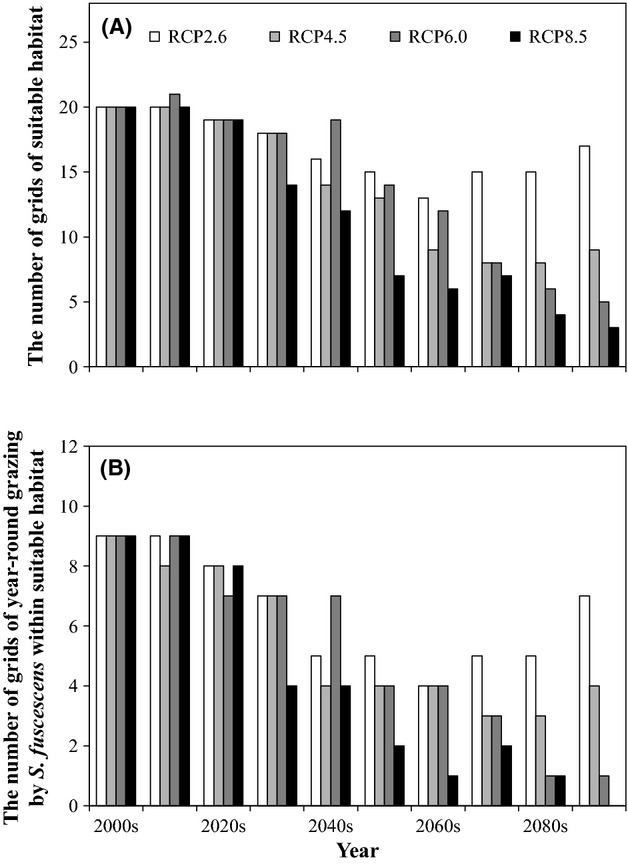
Shifts in the spatial extent of (A) suitable habitat and (B) year-round grazing by *S. fuscescens* within the suitable habitat between the 2000s and 2090s.

To evaluate the effects of grazing by *S. fuscescens* on the distribution of *E. cava* habitats under the warming scenarios, potential habitats were subdivided into four groups (Fig.1): suitable habitats without high-temperature stress or year-round grazing (blue squares); suitable habitats without high-temperature stress, but with year-round grazing (light blue squares); unsuitable habitats with high-temperature stress (yellow squares); and unsuitable habitats with high-temperature stress and year-round grazing (red squares). Within the regions offering suitable habitats for *E. cava*, the total area affected by year-round grazing was most extensive in the RCP2.6 scenario during the 2090s (Fig.1). In those areas (i.e., light blue squares in Fig.1), however, decadal mean SST in the warmest and coldest months by the 2090s was higher than 25°C and lower than 20°C, respectively (Figs S3–S8). In contrast, the year-round grazing-affected area in the RCP8.5 scenario declined gradually toward the end of the 21st century and was completely absent in the 2090s (Figs1 and 3). Regarding RCP4.5 and RCP6.0, the difference in the area affected by the year-round grazing area was relatively small before the 2070s, except during the 2040s (Fig3). For the RCP2.6 scenario, the combination of high-temperature stress and year-round grazing was the cause of most of the unsuitable habitat observed in the 2090s. For the remaining RCPs, unsuitable habitats in the Sea of Japan were primarily caused by high-temperature stress, whereas habitats unsuitability in the Pacific Ocean was caused by a combination of high-temperature stress and year-round grazing (Fig1c–e).

## Discussion

In this study, for the first time, distributional shifts in *E. cava* habitats around Japan were assessed in terms of rising water temperatures and grazing over the course of the 21st century. This analysis was based on multiple CMIP5 monthly SST projections forced with four RCP scenarios. Our projections for habitat change using these SST-based indices were in good agreement with the observed chronological changes in *E. cava* habitats from historical data (Table2). Although we used only SST-based indices in this study, our model performance is consistent when compared with previous studies, which evaluated the effects of climate change on seaweeds (e.g., Jueterbock et al. 2013). These results indicate that water temperature is a key factor in determining future suitable habitats for *E. cava*, and the presently available suitable habitat was well represented by our SST-based indices at a coarse horizontal resolution.

It is known that the species of *Ecklonia* has heteromorphic lift history (i.e., sporophytes and gametophytes phases), suggesting that optimal and tolerable temperatures for the growth might be different in the two phases. Thus, when SST-based indices for *E. cava* distribution are developed, we need to consider differences in those temperatures between the two phases. In sporophytes phase, Serisawa et al. (2001) reported that *E. cava* can grow at water temperatures ranged from 10 to 27°C and that optimal temperature ranges for the growth of the species were from 25 to 27°C. On the other hand, *E. cava* in gametophytes phase can grow at water temperatures ranged from 10 to 28°C (Ohta 1988; Tanaka et al. 2008), although there was a risk of developing abnormal gametophytes at 28°C (Tanaka et al. 2008). As a result, the overlapped temperature ranges for the growth of *E. cava* in each life history were from 10 to 27°C, which were consistent with our SST-based indices for *E. cava* distribution used in this study.

Our results suggest that continued warming may drive a poleward shift in the distribution of the temperate seaweed *E. cava,* with large differences depending on the severity of warming (Fig.1). For the RCPs 4.5, 6.0, and 8.5, potential suitable habitats were projected to move into northern Japanese coastal waters, where *E. cava* populations are not found currently. Although it is unknown why *E. cava* is absent from this region, it is possible that the warm Kuroshio and cold Oyashio currents may limit its distribution. The Kuroshio Current flows northward along the south coast of Japan and transports not only warm and saline surface waters (e.g., Yasuda 2003) but also seaweed spores and coral larva from south to north (e.g., Veron and Minchin 1992). On the other hand, the Oyashio Current flows southward along the east coast of Japan. These two currents converge in the area east of Japan, and then, the Kuroshio Current, potentially with subtropical and temperate seaweed spores, recedes from the east coast of Japan as the Kuroshio Extension. Thus, if existing *E. cava* populations cannot in fact affect the projected northward shift, they may become extinct due to high-temperature stress and/or grazing by herbivores, as projected in the higher emission scenarios described here (Fig.1). Furthermore, marine organisms associated with *E. cava*, such as endemic abalone, may also be under threat of extinction, and consequently, Japanese fisheries and livelihoods dependent on *E. cava* may suffer heavy damage. Although a shift in the distribution of a single species is in itself serious, changes in entire communities related to these species can have negative impacts on ecosystem function that propagate upward through the trophic web.

For the RCP2.6 scenario, our results suggest that most existing *E. cava* populations would survive the resulting high-temperature stress (Figs1 and 2), while at the same time being exposed to increased grazing by the herbivorous fish *S. fuscescens* (Figs1 and 3). However, under RCP4.5, the size of the suitable habitat is expected to decrease by 45% by the 2090s, compared with the present, and more than half of that area may be impacted by year-round grazing (i.e., light blue squares in Fig.1). Our projections suggest that if *E. cava* is not protected from herbivores*,* grazing would further limit the utilization of the suitable habitat, even if it were possible to mitigate regional warming. These results strongly emphasize the importance of protecting *E. cava* from herbivores under the lower emission scenarios. Several means to protect *E. cava* from herbivores, such as net cages and spore-bag techniques, have been investigated in the literature (e.g., Okuda 2008; Haraguchi et al. 2009). Furthermore, the commercial exploitation of herbivorous fishes has been attempted in local towns, where they sell *S. fuscescens* as a dried food product. However, given that there is no highly effective or efficient means to protect *E. cava* thus far (Fisheries Agency of Japan 2007), the population control of herbivores and further improvement of protection techniques are required to conserve existing *E. cava* populations. However, our results also show that even if *E. cava* populations are protected from herbivores, ongoing warming in the high-emissions scenario (RCP 8.5) may still devastate the species in Japanese coastal waters (Fig.1). In April 2014, the globally averaged mole fraction for CO_2_ reached 398.87 ppm (http://www.esrl.noaa.gov/gmd/ccgg/trends), which is in fact higher than the global emissions trajectories assumed in 2014 for RCPs 2.6–6.0 and slightly lower than that for RCP8.5. Therefore, to conserve *E. cava* populations around Japan, it will be necessary to both mitigate seawater warming, as well as protect *E. cava* from herbivorous fishes.

Several limitations and assumptions inherent to this study may lead to uncertainties in our projections. The threshold values used to project habitat availability in this study were based on statistical results compared with the observed presence of *E. cava* (Table2), but did not include physiological considerations, such as acclimation or adaptation to thermal stress. Although adaptation to thermal stress has not been studied extensively in seaweeds, several studies have reported seaweed acclimation to high-temperature conditions (Harley et al. 2012 and references therein). For example, some warm-acclimated seaweed species were more efficient photosynthetically at high temperatures and more likely to survive periods of thermal stress (Davison et al. 1991; Li and Brawley 2004). Furthermore, some studies have reported that it is possible to recover from the near-complete loss of seaweeds (e.g., Edwards 2004; Fisheries Agency of Japan 2007). Although the recovery rate varies considerably according to several factors (e.g., location, species, and growth conditions) and has not been examined quantitatively (e.g., Edwards 2004; Tanaka et al. 2012), integrating such recovery processes might lead to more accurate and realistic projections. Therefore, a better understanding of the extent to which *E. cava* can adapt to thermal stress and/or recover from near-complete elimination is crucial for predicting its future habitats.

Water temperature is an important determinant of seaweed survival, growth, and reproduction, as are CO_2_ concentrations and related changes in ocean chemistry, such as acidification (Harley et al. 2012 and references therein). Several studies have evaluated the combined impacts of warming and ocean acidification on other habitat-forming species, such as coral reefs, using climate model projections (e.g., Yara et al. 2012; van Hooidonk et al. 2014). However, no such study has yet been conducted for seaweeds, equally important ecological engineers. In addition to the effects of warming, further studies examining the effects of other environmental factors on seaweeds would lead to more accurate and realistic projections of *E. cava* habitat availability.

This study presents the first projections to consider the interspecific interaction between seaweeds and herbivores based on outputs of the latest climate models forced with the most up-to-date future emissions scenarios. Whereas previous studies using climate model projections have focused solely on the direct effects of rising water temperatures on seaweeds, we also considered the effects of grazing by the herbivorous fish *S. fuscescens* on the distribution of habitats suitable for the temperate seaweed *E. cava* under several emissions scenarios. Our projections suggest that continued warming may drive a poleward shift in *E. cava* populations*,* with large differences depending on the scenario examined. Furthermore, even if regional warming can be mitigated, most *E. cava* populations would still be exposed to increased grazing by herbivores. Therefore, our ability to preserve existing *E. cava* populations depends on the implementation of a range of measures aimed at both protecting habitats from herbivores as well as the mitigation of greenhouse gas emissions. Historical records regarding the distribution of seaweeds collected in this study will be useful in evaluating the adequacy of simulation-based studies. Furthermore, because seaweeds around Japan may be faced with more rapid warming relative to those of the world's oceans, this dataset could serve as a baseline for assessing the consequences of rapidly warming seas.

## Supporting Information

Table S1. Summary of the numbers of records for the 20 grid cells used to validate the estimated potential habitats for *Ecklonia cava*.

Data S1. References.

Figure S1. Site locations in which the observed and projected results were in agreement (gray squares).

Figure S2. Shifts in the decadal mean SST in (A) the warmest and (B) coldest months between the 2000s and 2090s.

Figure S3. Decadal mean SST in the warmest months using the CMIP5 multimodel mean monthly SSTs in (A) the 2000s and (B–E) the 2030s for RCPs 2.6, 4.5, 6.0, and 8.5, respectively.

Figure S4. Same as Figure S3 but in the 2060s.

Figure S5. Same as Figure S3 but in the 2090s.

Figure S6. Decadal mean SST in the coldest months using the CMIP5 multimodel mean monthly SSTs in (A) the 2000s and (B–E) the 2030s for RCPs 2.6, 4.5, 6.0, and 8.5, respectively.

Figure S7. Same as Figure S6 but in the 2060s.

Figure S8. Same as Figure S6 but in the 2090s.
