# Supplementary material for: Projecting the impacts of rising seawater temperatures on the distribution of seaweeds around Japan under multiple climate change scenarios
Source: Ecol Evol. 2014 Dec 18;5(1):213–23. doi: 10.1002/ece3.1358 (PMC4298448; doi:10.1002/ece3.1358)
Supplement: Supplementary file 1 [file ece30005-0213-sd1.pdf]

Latitude (°)	Longitude (°)	1960s	1970s	1980s	1990s	2000s
37.5	137	2/2	n.d.	n.d.	6/6	n.d.
36.5	136	n.d.	n.d.	n.d.	2/2	n.d.
35.5	133	n.d.	5/5	4/4	1/1	n.d.
35.5	134	3/4	n.d.	3/3	n.d.	1/1
34.5	131	n.d.	20/20	n.d.	n.d.	1/1
34.5	135	1/1	1/1	5/5	5/5	4/4
34.5	138	1/1	n.d.	1/1	1/2	1/3
34.5	139	2/2	47/47	1/1	7/14	4/8
34.5	140	n.d.	2/2	1/1	1/1	1/1
33.5	129	n.d.	4/11	n.d.	4/4	0/1
33.5	130	5/5	15/15	13/13	2/2	13/20
33.5	135	5/9	7/7	8/12	3/3	16/27
32.5	129	n.d.	1/4	n.d.	1/6	n.d.
32.5	130	2/2	7/9	2/2	2/2	4/14
32.5	133	n.d.	3/3	4/4	2/4	2/5
32.5	134	n.d.	2/2	5/5	6/6	1/6
31.5	130	n.d.	0/1	n.d.	n.d.	0/2
31.5	132	1/1	n.d.	3/3	1/3	2/5
30.5	130	n.d.	n.d.	n.d.	n.d.	0/1
30.5	131	n.d.	n.d.	n.d.	n.d.	0/1

Numbers indicate the ratio of presence records of *E. cava* to total records. Gray colors indicate the grid cell assigned as presence of *E. cava*, including “possibly presence” assigned via the temporal and/or spatial interpolation.
