# Supplementary material for: Projecting the impacts of rising seawater temperatures on the distribution of seaweeds around Japan under multiple climate change scenarios
Source: Ecol Evol. 2014 Dec 18;5(1):213–23. doi: 10.1002/ece3.1358 (PMC4298448; doi:10.1002/ece3.1358)
Supplement: Supplementary file 2 [file ece30005-0213-sd2.docx]

**Data S1 References**

Akimoto T, Nakamoto T, Watanabe D, Futigami S, Tsukushi Y (2008) Changes and present status of the macrophyte beds in Chikuzen Sea. *Bulletin of Fukuoka Fisheries and Marine Technology Research Center*, **18**, 65–75 (in Japanese).

Chiba Prefecture (1971) Academic Investigation Report of Marine Park in Chiba Prefecture. Pp. 89 (in Japanese).

Construction Engineering Research Institute (1966) Total Survey Report on the Sea Floor in Sanin Marine Park. Pp. 123 (in Japanese).

Environment Agency (1994) The Report of the Marine Biotic Environment Survey in the 4th National Survey on the Natural Environment. Vol. 2 Algal and Sea-grass Beds (in Japanese, without page number).

Hasegawa M (1996) Unusual event on the seaweed *Eisenia arborea*. *Letters in Izu Branch of Shizuoka Prefectural Fisheries Experiment Station*, **264**, 2–8 (in Japanese).

Hayashida F, Sakurai T (1969) Algal flora and communities at Mochimune, Suruga Bay. *Japanese Journal of Ecology*, **19**, 52–56 (in Japanese with English abstract).

Hayashida F (1984) Potential production of the aquatic forest-forming brown algal, *Ecklonia cava* Kjellman, calculated from individual year classes. *Hydrobiologia*, **116/117**, 429–432.

Ikemori T, Tajima M (2002) List of marine plants from the coast of Ishikawa Prefecture. *Bulletin of Ishikawa Prefectural Fisheries Research Center*, **3**, 1–11 (in Japanese and English abstract).

Ishida K, Yuuki Y (1996) Seasonal changes of *Ecklonia kurome* Okamura off Kashima, Shimane Prefecture. *The Aquaculture*, **44**, 241–247 (in Japanese with English abstract).

Ishikawa Prefecture (1970) Academic Investigation Report of Marine Park in Noto Peninsula, Ishikawa Prefecture. Pp. 127 (in Japanese).

Iwahashi Y, Inaba S, Fushimi H, Sasaki T, Osuga H (1979) Ecological studies on Eisenia and Ecklonia in the coast of Izu Peninsula - IV. The distribution and characteristics of kelp stand. *Bulletin of the Shizuoka Prefectural Fisheries Experiment Station*, **13**, 75–82 (in Japanese).

Kiriyama T (2009) Study on recent decline of large brown alga population in coastal waters around Nagasaki Prefecture. *Bulletin of Nagasaki Prefectural Institute of Fisheries*, **35**, 15–78 (in Japanese).

Kitayama T (1998) Brown algae from Fukue Island, Nagasaki Pref., Japan. *Memoirs of the National Science Museum, Tokyo*, **31**, 98–106.

Konishi Y, Hayashida F (2004) Vegetation of benthic marine algae in Suruga Bay, Central Japan. *Journal of The School of Marine Science and Technology*, **11**, 15–27 (in Japanese with English abstract).

Kumamoto Prefecture (1968) Academic Investigation Report of Marine Park in Kumamoto Prefecture. Pp. 98 (in Japanese).

Kushimoto Marine Park Center (2010) Investigation Report of Coral Distribution in Wakayama Prefecture. Pp. 136 (in Japanese).

Matsui T, Ohgai M, Ouchi T, Kakuda N, Nakamura T (1984) Marine algal vegetation around the coast of the middle part of Yamaguchi prefecture along the Japan Sea. Journal of Shimonoseki University of Fisheries, 32, 91–113 (in Japanese with English abstract).

Ministry of the Environment (2008) The Report of the Coastal Ecosystem Survey in the 7th National Survey on the Natural Environment (Algal and sea-grass beds). Pp. 433 (in Japanese).

Nagasaki Prefecture (1971) Academic Investigation Report of Marine Park in Nagasaki Prefecture. Pp. 116 (in Japanese).

Nagasaki Prefecture (1975) Academic Investigation Report of Marine Park in Kami Goto Islands, Hirado, Kujyukushima Islands, Nagasaki Prefecture. Pp. 101 (in Japanese).

Nagasaki Prefecture (2012) Guideline for Management of Barren Grounds on the Sea Floor in Nagasaki Prefecture. Pp. 73 (in Japanese).

Nakahisa Y, Tanimoto N, Kojima H (1976) Report on the algal flora survey along Kaifu Coast 1. *Annual Report of Tokushima Prefectural Fisheries Research Center* 98–102 (in Japanese).

Nakanishi T (2009) Ongoing drastic changes of the macrophyte beds along Kaifu Coast. *Letters in Tokushima Prefectural Fisheries Research Center*, **71**, 1–7 (in Japanese).

Narihara J, Terawaki T (1992) A community of *Ecklonia kurome* on the coastal structures in Kawaminami Fishing Port, Miyazaki Prefecture. *The Aquaculture*, **40**, 173–175 (in Japanese with English abstract).

Saga Prefecture (1969) Academic investigation report of marine park in Saga Prefecture. Pp. 50 (in Japanese).

Serisawa Y, Takaki H, Kurashima A, Yokohama Y (2001) Seasonal change in nitrate concentration in seawater and photosynthetic activity of bladelets of *Ecklonia cava* in Nabeta Bay, Shimoda, southern part of Izu Peninsula, Japan. Nippon Suisan Gakkaishi, 67, 1065–1071 (in Japanese with English abstract).

Shimane Prefecture (1971) Academic Investigation Report of Marine Park in Hinomisaki Headland, Shimane Prefecture. Pp. 100 (in Japanese).

Tanaka K, Taino S, Haraguchi H, Prendergast G, Hiraoka M (2012) Warming off southwestern Japan linked to distributional shifts of subtidal canopy-forming seaweeds. *Ecology and Evolution*, **2**, 2854–2865.

Tanaka T, Sakamoto J, Ikegami N, Hirata J (2002) On the community of a brown alga, *Ecklonia cava* in the Sotobo coastal zone, Chiba Prefecture. *Bulletin of the Chiba Prefectural Fisheries Research Center*, **1**, 51–59 (in Japanese).

Tashiro K (2010) Seaweeds on the coast of Aosima, Miyazaki, Miyazaki Prefecture, Japan. *Bulletin of Miyazaki Prefectural Fisheries Experimental Station*, **12**, 5–13 (in Japanese).

Terada R, Kawai H, Kurashima A, Murase N, Sakanishi Y, Tanaka J, Yoshida G, Abe T, Kitayama T (2013) Distribution of the five major Japanese laminarian species as an appropriate indicator of global changes by long-term monitoring. In: *Survey report 2008–2012 of the coasts and shallow seas in the Monitoring Sites 1000: rocky shores, tidal flats, seagrass beds and seaweed beds*, Biodiversity Center, Ministry of the Environment, Japan. Pp. 68–73 (in Japanese).

Terawaki T, Goto H, Honda M (1991) Verification of Technologies for Kelp Forest Creation on Sandy Sea Beds. I. Literature and Case Surveys on Techniques for Kelp Forest Creation. *CRIEPI Research Report*, U91021, Pp. 38 (in Japanese with English abstract).

Terawaki T, Kawasaki Y (1990) Development of Techniques for Marine Macrophyte Bed Creation on Sandy Sea Beds. III. Growth of *Ecklonia kurome* Plant and its Limiting Factors in Natural Sea Beds. *CRIEPI Research Report*, U90044, Pp. 25 (in Japanese and English abstract).

Terawaki T, Yoshikawa K, Yoshida G, Uchimura M, Arai S (2002) Seascape features of "Isoyake Area" in Southeast Japan. *Fisheries Engineering*, **39**, 29–35 (in Japanese with English abstract).

Wakayama Prefecture (1966) Academic Investigation Report of Marine Park in Wakayama Prefecture. Pp. 127 (in Japanese).

Wakayama Prefecture (1973) Academic Investigation Report of Marine Park in Kumano Karekinada Coast, Wakayama Prefecture. Pp. 33 (in Japanese).
