# Supplementary figures and images for: Projecting the impacts of rising seawater temperatures on the distribution of seaweeds around Japan under multiple climate change scenarios
Source: Ecol Evol. 2014 Dec 18;5(1):213–23. doi: 10.1002/ece3.1358 (PMC4298448; doi:10.1002/ece3.1358)

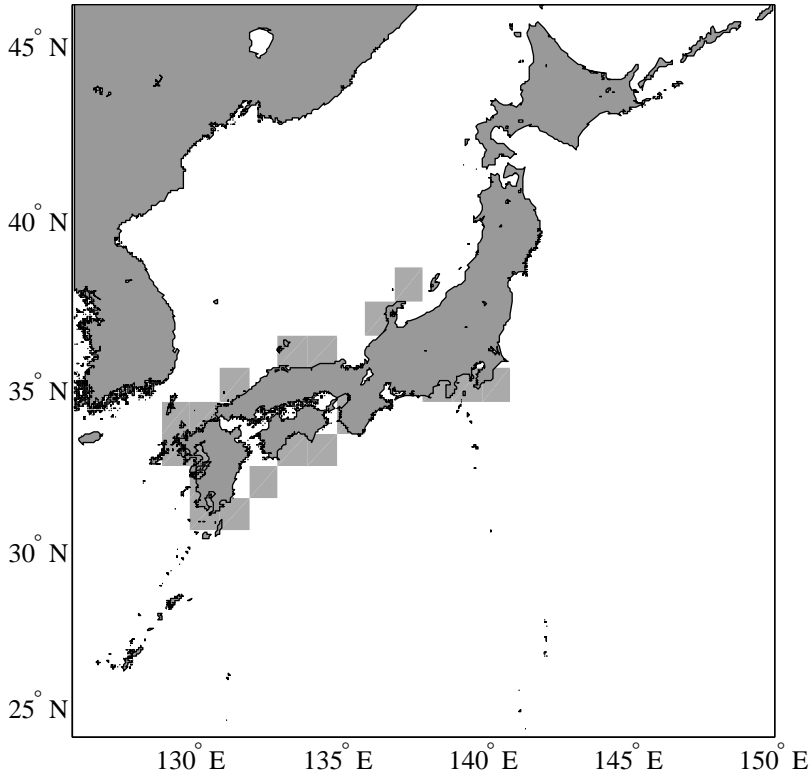

Supplement: Supplementary file 3 [file ece30005-0213-sd3.pdf]

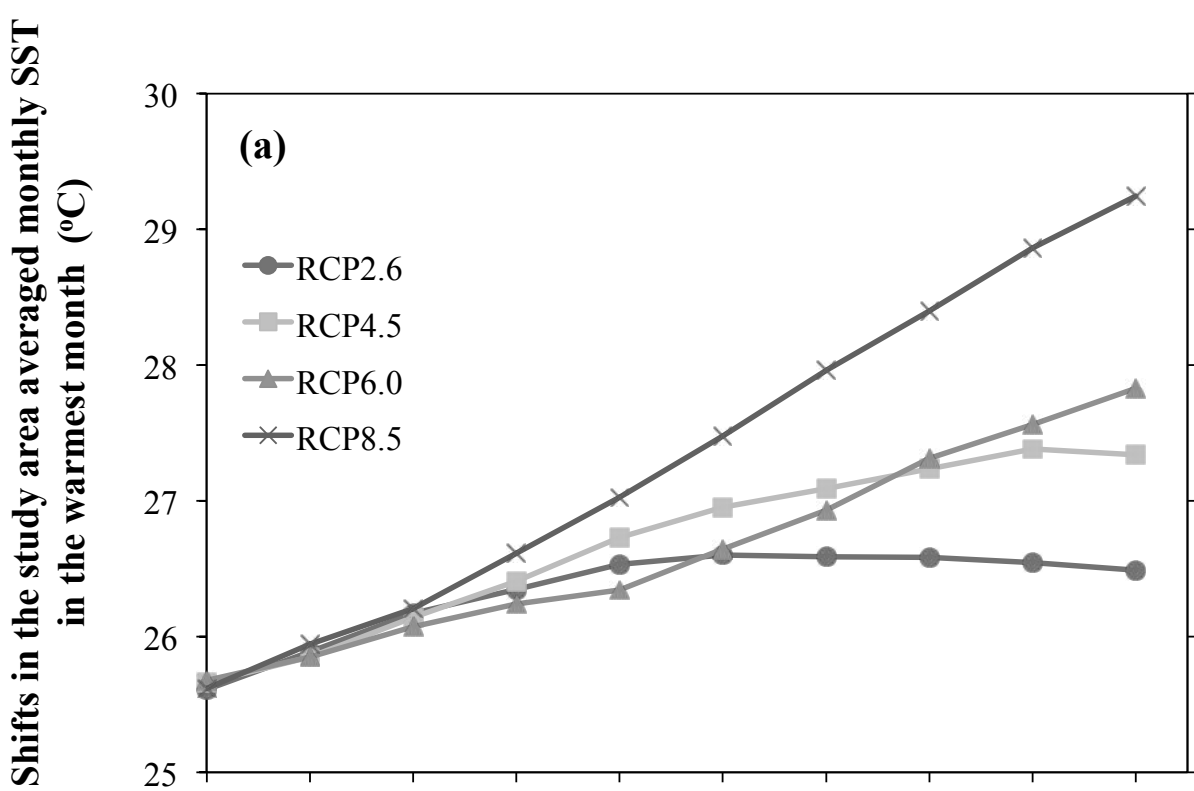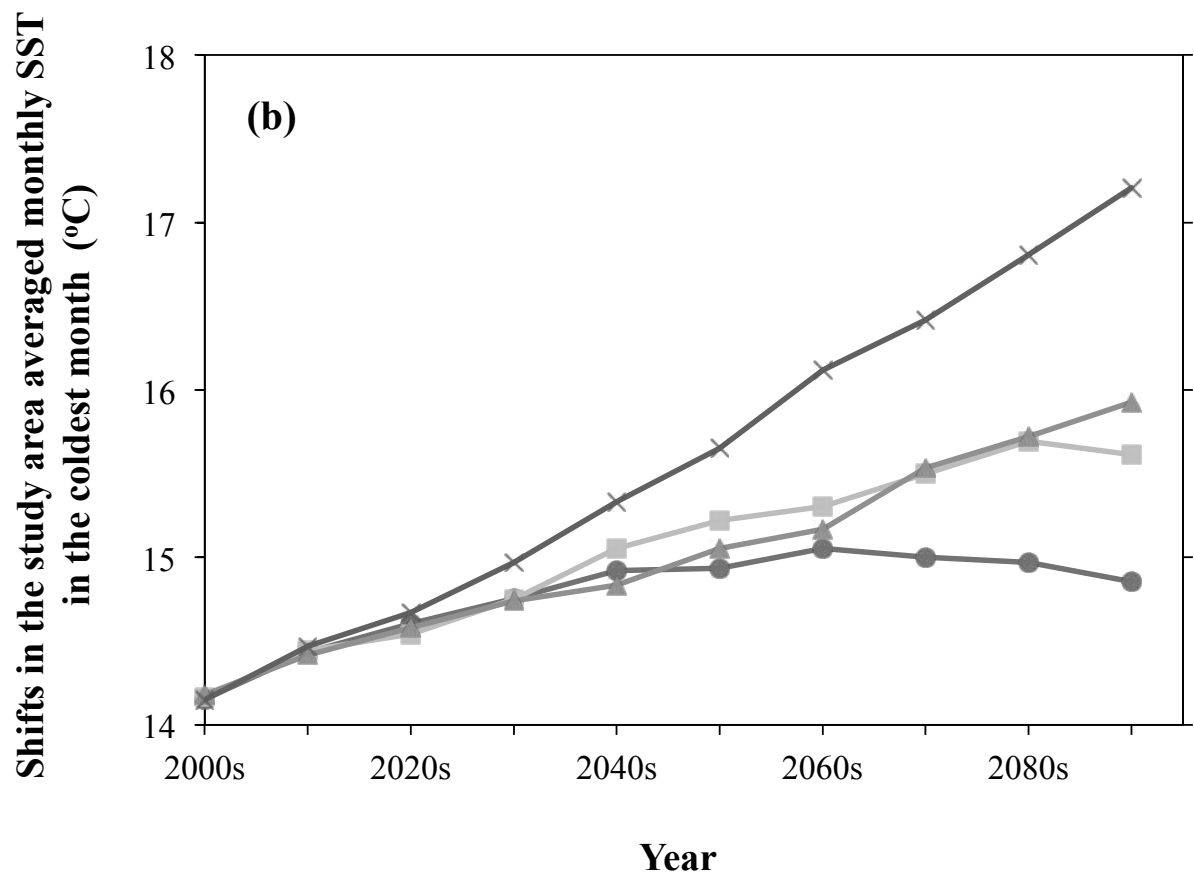

Supplement: Supplementary file 4 [file ece30005-0213-sd4.pdf]

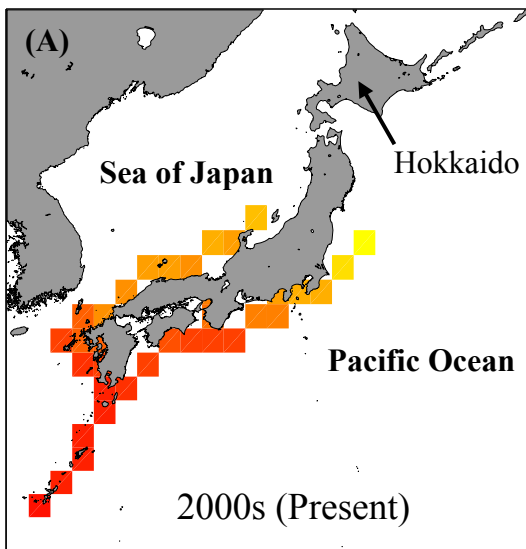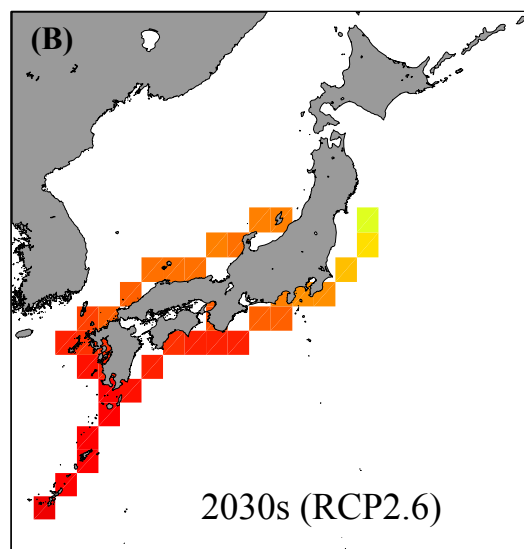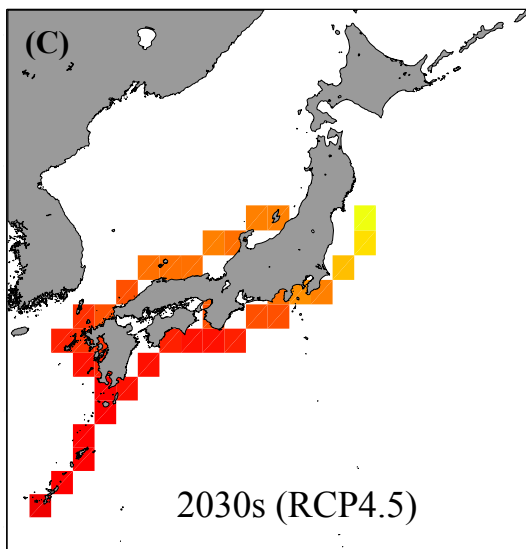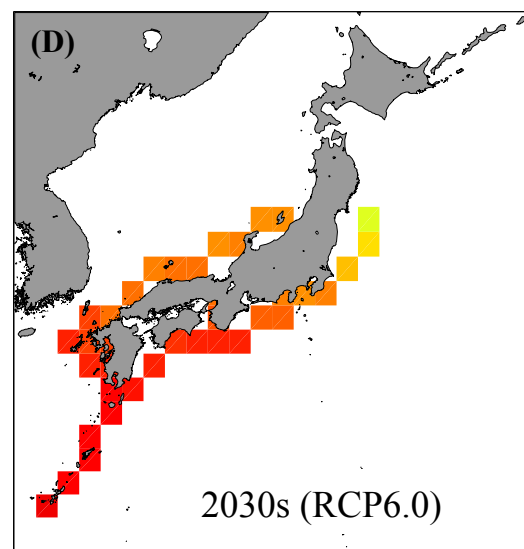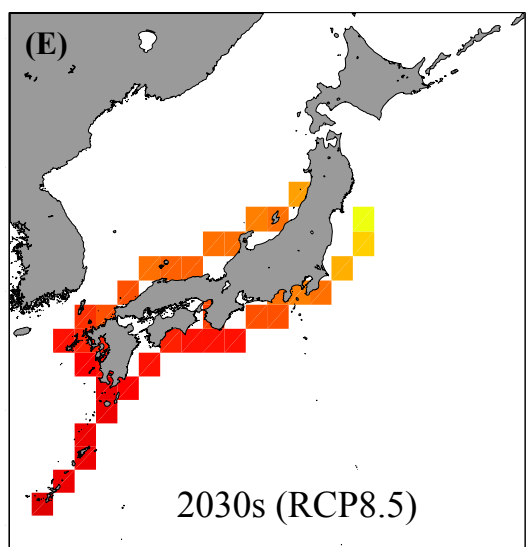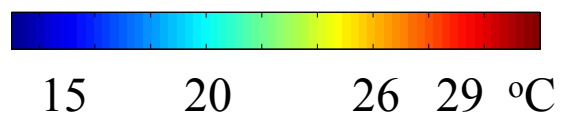

Supplement: Supplementary file 5 [file ece30005-0213-sd5.pdf]

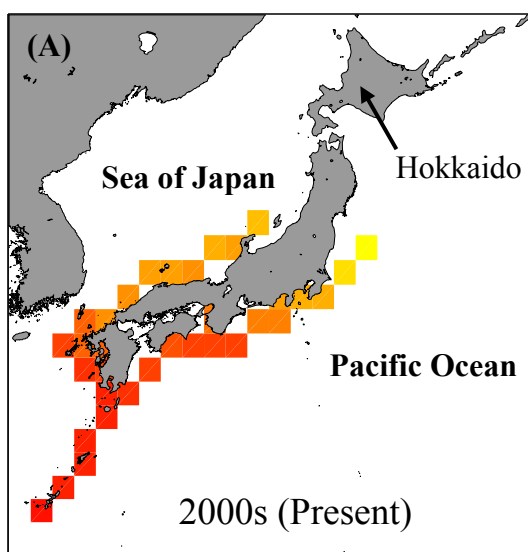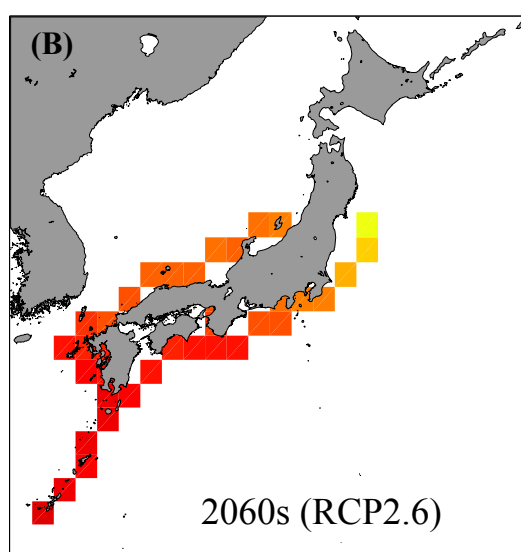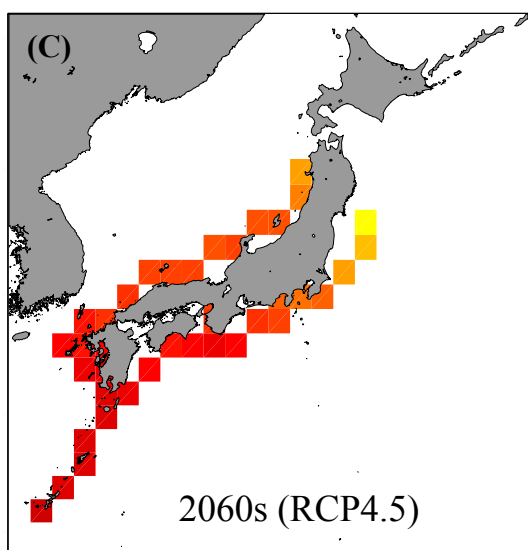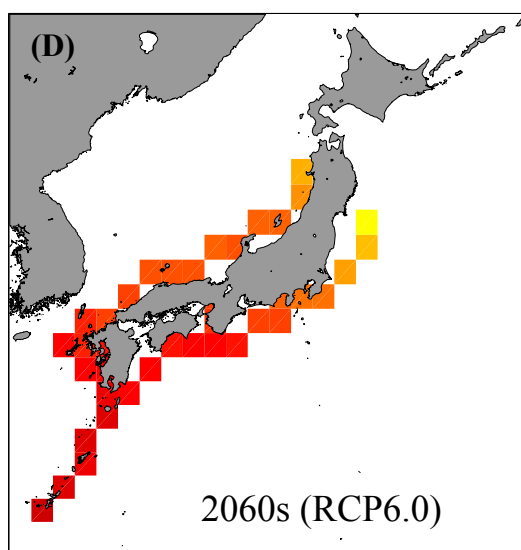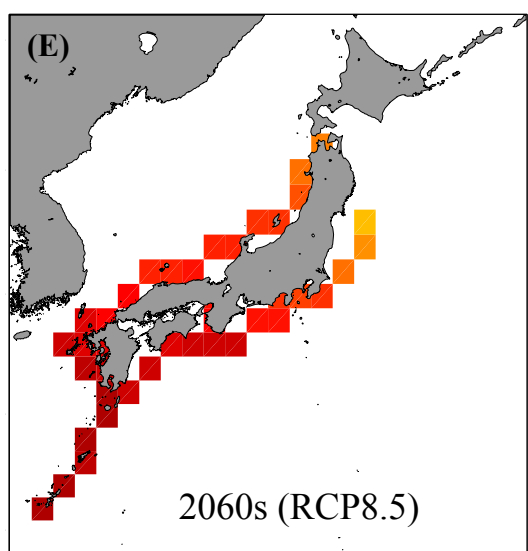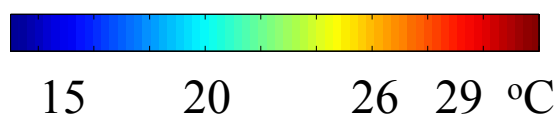

Supplement: Supplementary file 6 [file ece30005-0213-sd6.pdf]

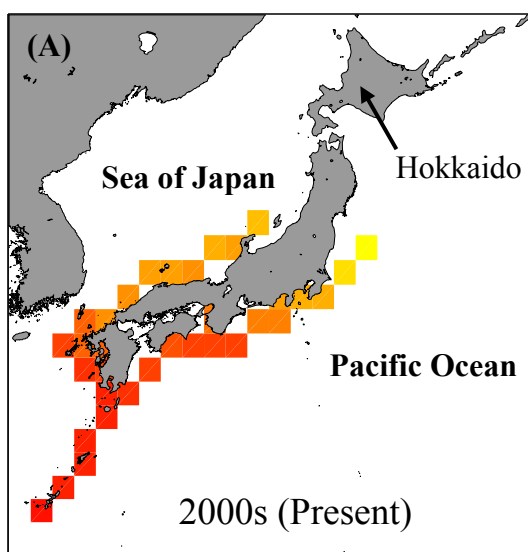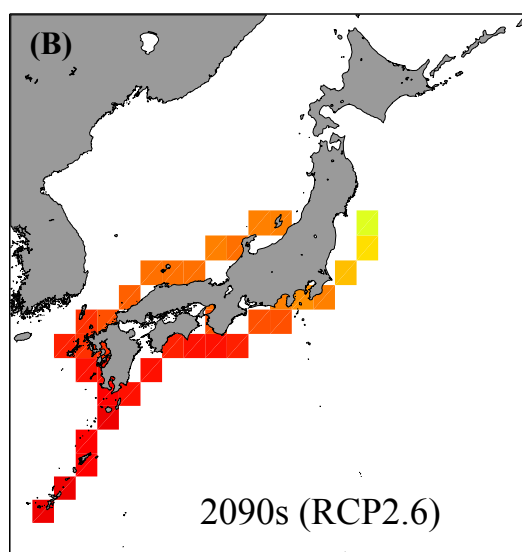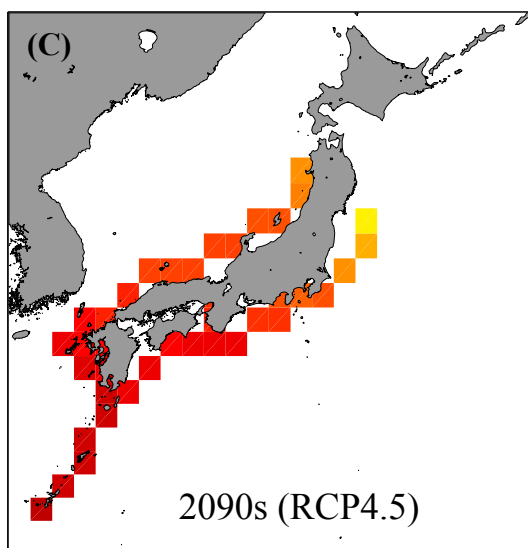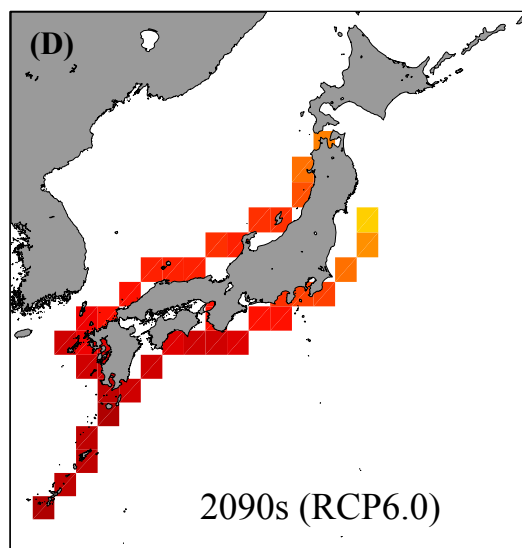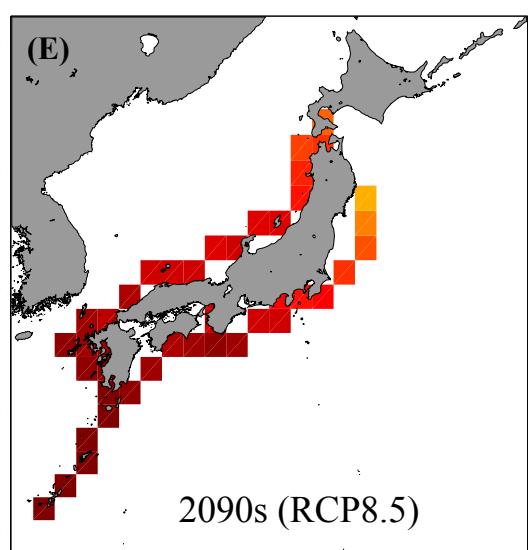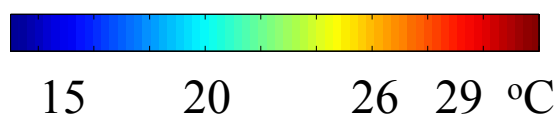

Supplement: Supplementary file 7 [file ece30005-0213-sd7.pdf]

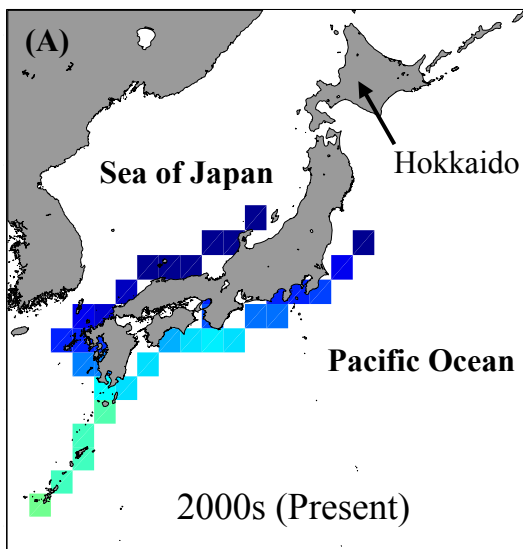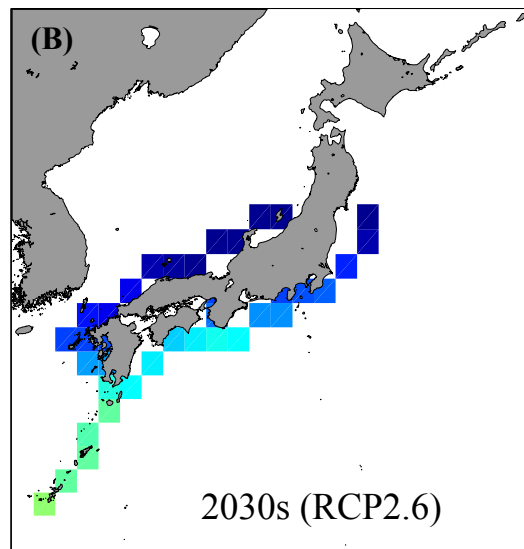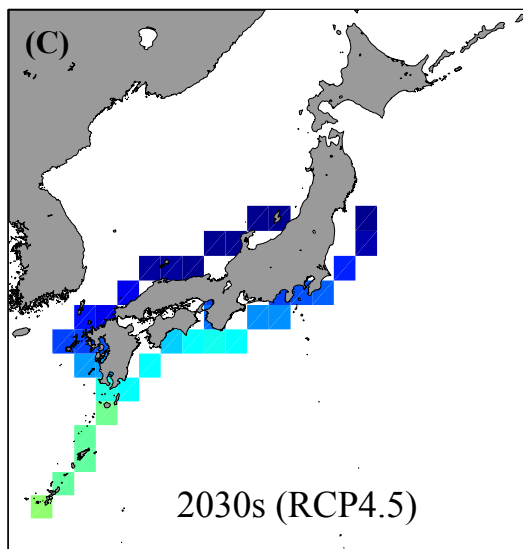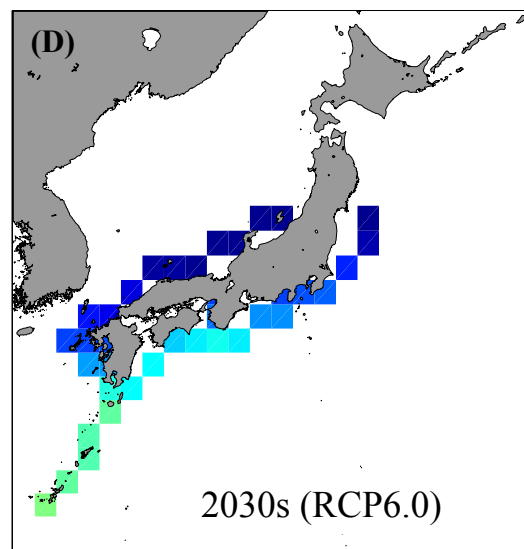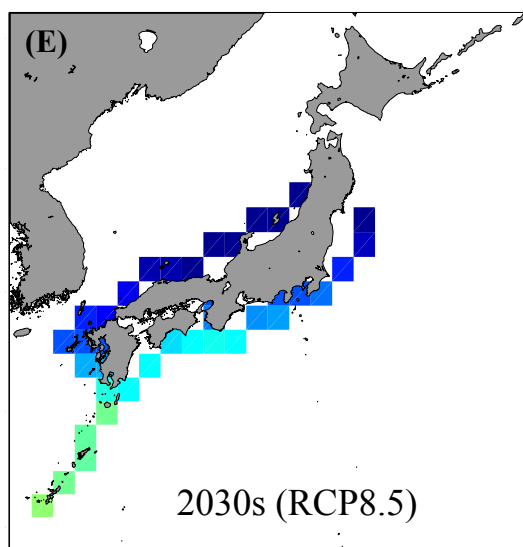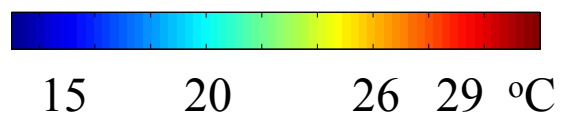

Supplement: Supplementary file 8 [file ece30005-0213-sd8.pdf]

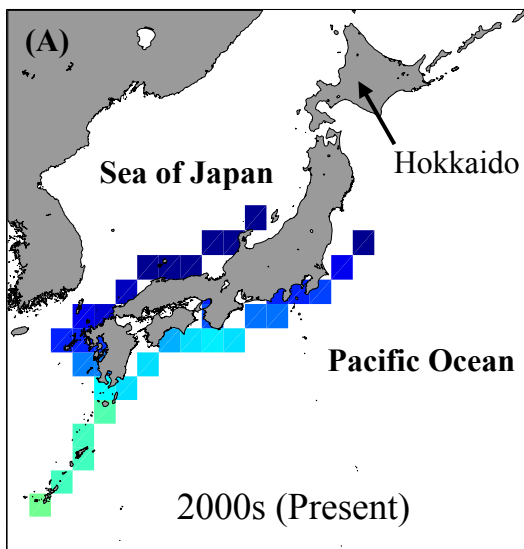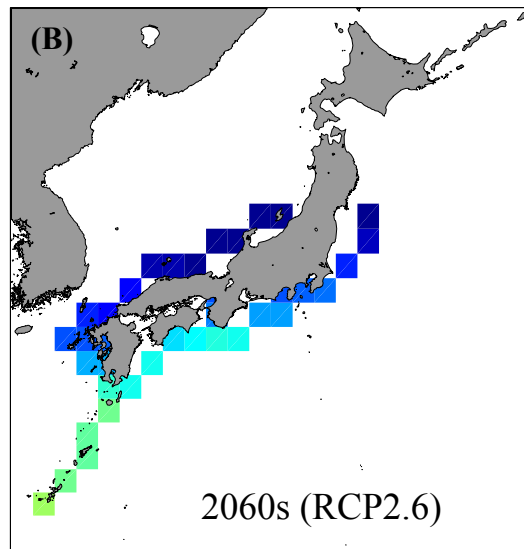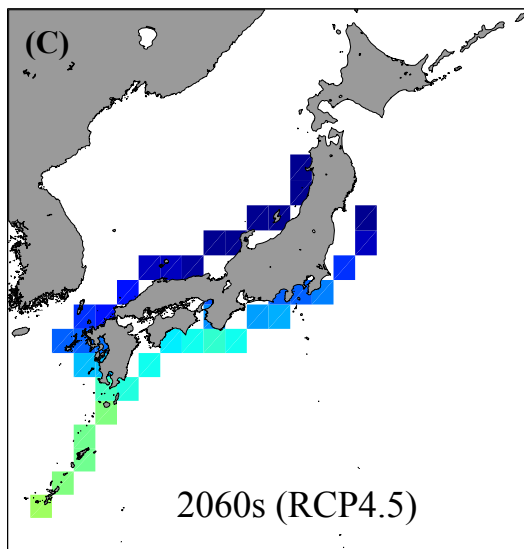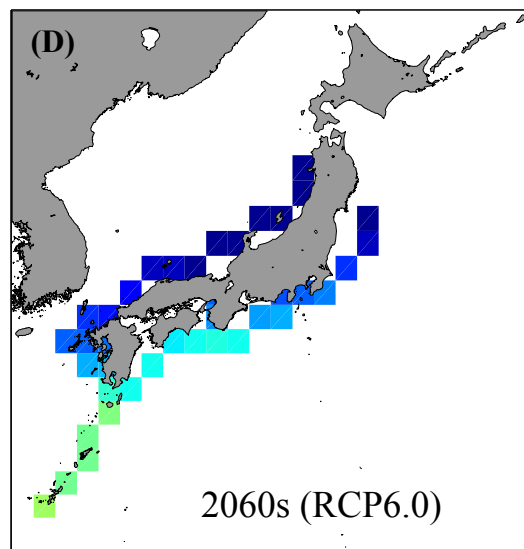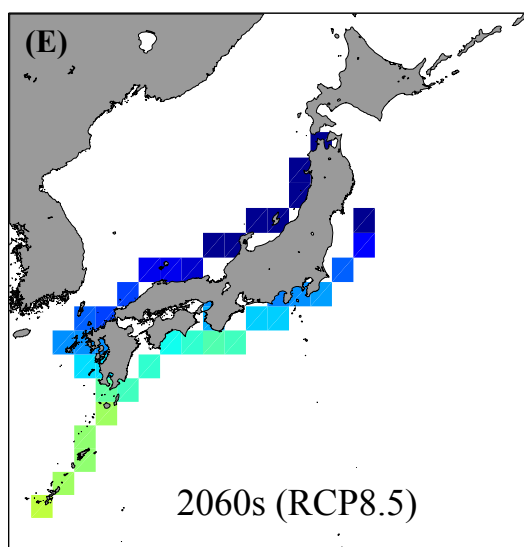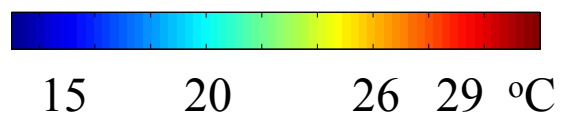

Supplement: Supplementary file 9 [file ece30005-0213-sd9.pdf]

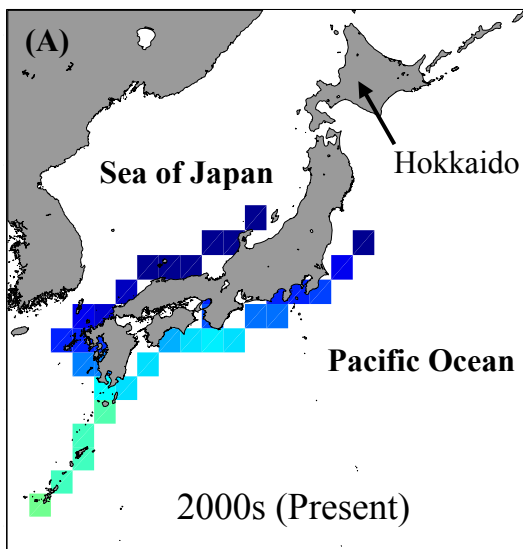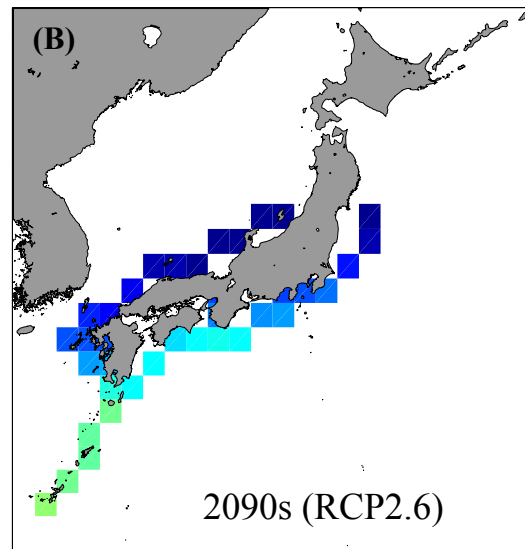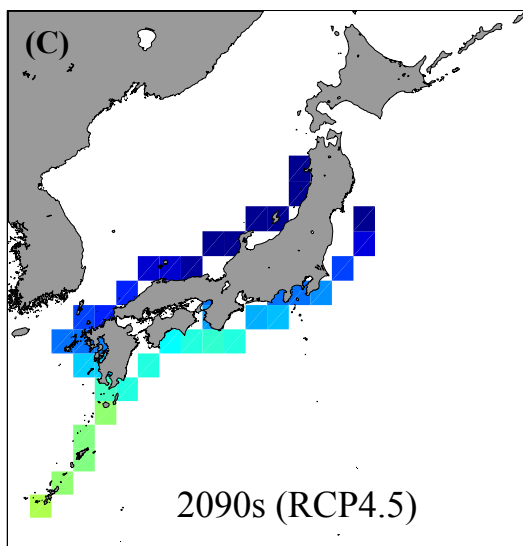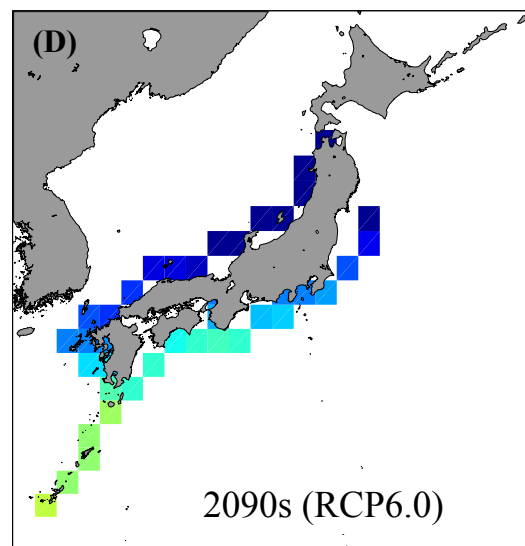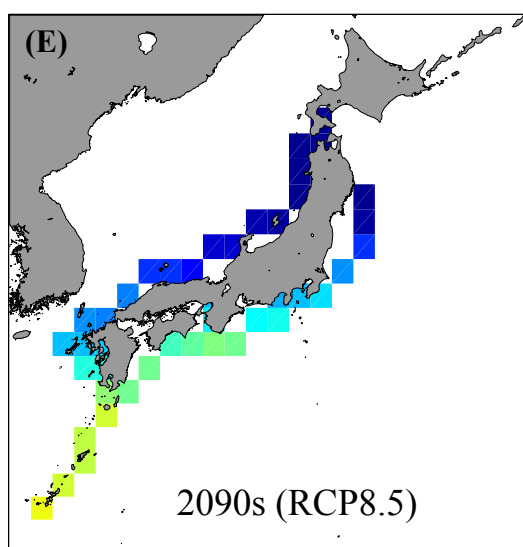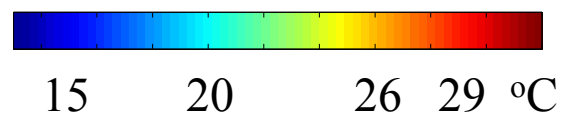

Supplement: Supplementary file 10 [file ece30005-0213-sd10.pdf]
